# Supplementary material for: Altered microRNA Transcriptome in Cultured Human Liver Cells upon Infection with Ebola Virus
Source: Int J Mol Sci. 2021 Apr 6;22(7):3792. doi: 10.3390/ijms22073792 (PMC8038836; doi:10.3390/ijms22073792)
Supplement: Supplementary file 1 [file ijms-22-03792-s001.zip › Supplementary_File/D_ KEGG_Analysis_Results/16-30nt_pathway_Makona-96h-Huh7_vs_Control-96h-Huh7_down.mature_mirna_targets/hsa_GeneRatioDotPlot.pdf]

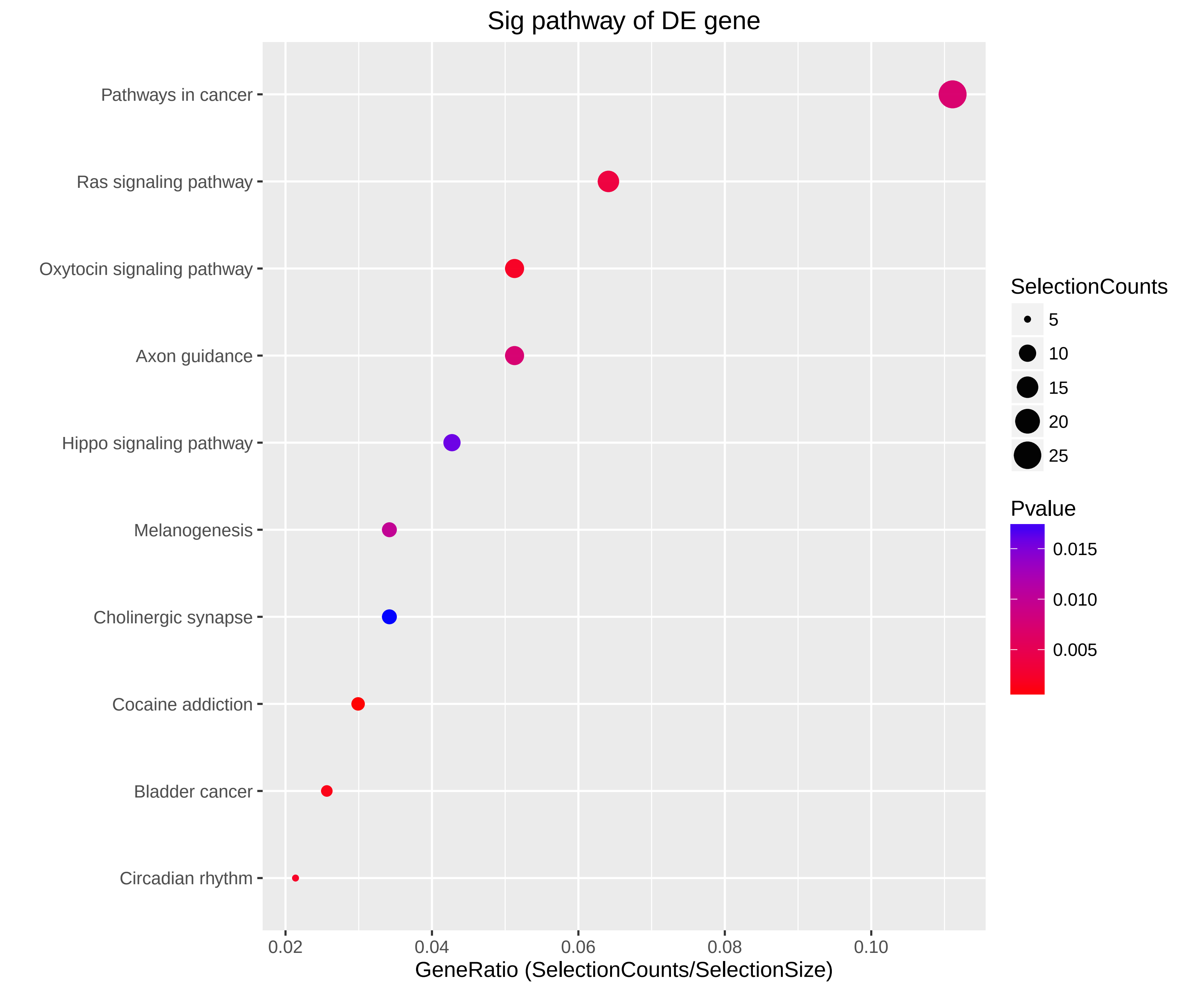
Sig pathway of DE gene
Pathways in cancer
Ras signaling pathway
Oxytocin signaling pathway
Axon guidance
Hippo signaling pathway
Melanogenesis
Cholinergic synapse
Cocaine addiction
Bladder cancer
Circadian rhythm
0.02
0.04
0.06
0.08
0.10
GeneRatio (SelectionCounts/SelectionSize)
SelectionCounts
5
10
15
20
25
Pvalue
0.015
0.010
0.005
